# Voxel-Wise Motion Artifacts in Population-Level Whole-Brain Connectivity Analysis of Resting-State fMRI

**DOI:** 10.1371/journal.pone.0104947

**Published:** 2014-09-04

**Authors:** Tamás Spisák, András Jakab, Sándor A. Kis, Gábor Opposits, Csaba Aranyi, Ervin Berényi, Miklós Emri

**Affiliations:** 1 Department of Nuclear Medicine, Medical and Health Science Centre, University of Debrecen, Debrecen, Hungary; 2 Department of Radiology, Medical University of Vienna, Vienna, Austria; 3 Department of Biomedical Laboratory and Imaging Science, Medical and Health Science Center, University of Debrecen, Debrecen, Hungary; Laureate Institute for Brain Research and The University of Oklahoma, United States of America

## Abstract

Functional Magnetic Resonance Imaging (fMRI) based brain connectivity analysis maps the functional networks of the brain by estimating the degree of synchronous neuronal activity between brain regions. Recent studies have demonstrated that “resting-state” fMRI-based brain connectivity conclusions may be erroneous when motion artifacts have a differential effect on fMRI BOLD signals for between group comparisons. A potential explanation could be that in-scanner displacement, due to rotational components, is not spatially constant in the whole brain. However, this localized nature of motion artifacts is poorly understood and is rarely considered in brain connectivity studies. In this study, we initially demonstrate the local correspondence between head displacement and the changes in the resting-state fMRI BOLD signal. Than, we investigate how connectivity strength is affected by the population-level variation in the spatial pattern of regional displacement. We introduce Regional Displacement Interaction (RDI), a new covariate parameter set for second-level connectivity analysis and demonstrate its effectiveness in reducing motion related confounds in comparisons of groups with different voxel-vise displacement pattern and preprocessed using various nuisance regression methods. The effect of using RDI as second-level covariate is than demonstrated in autism-related group comparisons. The relationship between the proposed method and some of the prevailing subject-level nuisance regression techniques is evaluated. Our results show that, depending on experimental design, treating in-scanner head motion as a global confound may not be appropriate. The degree of displacement is highly variable among various brain regions, both within and between subjects. These regional differences bias correlation-based measures of brain connectivity. The inclusion of the proposed second-level covariate into the analysis successfully reduces artifactual motion-related group differences and preserves real neuronal differences, as demonstrated by the autism-related comparisons.

## Introduction

### Background

Motion artifacts are problematic for all types of MRI including resting-state functional MRI (fMRI) and therefore motion correction is a vital step in every work-flow during fMRI analysis. According to population-level analysis and group comparisons, retrospective motion related artifact removal strategies can be performed at five different stages of the data processing pipeline: (i) motion correction of fMRI time-series by realignment to a reference image (using automatic co-registration approaches) [1]; (ii) censoring data to exclude periods of high motion (scrubbing, de-spiking) [2], [3]; (iii) modeling the effect of motion-related nuisance parameters on blood oxygen level dependent (BOLD) signal [4]–[7]; (iv) temporal filtering of BOLD timecourses to discard frequencies encumbered by motion artifacts and (v) correct for subject-specific motion effects on population-level (descriptive summary statistics of subject-specific motion as second-level model regressors) [8]–[10].

Traditional realignment-based correction approaches ensure that different time-points of the BOLD signal correspond to the same location. However, such methods do not handle voxel-level intensity confounds originating from the establishment of magnetic gradients and subsequent readout of the BOLD signal [2], [11]. Furthermore, automatic co-registration approaches may introduce spurious displacements in intervals of relatively low motion [12]. Nonetheless, subject movement is often measured with parameters based upon the resulting image realignment transformations.

Large BOLD intensity confounds (spikes) in time-frames with extreme, abrupt movement can be eliminated from the analysis by simply dropping the corrupted data (“scrubbing”) [2] or by spike-regression [13]. However, the reduction in time points is associated with an increase in the likelihood of high correlation scores [8]; moreover, recent findings [8], [14], [15] suggest that in population-level functional connectivity studies, scrubbing can be omitted from the analysis when using proper second-level correction.

Intensity confounds originating from micro-movements (as small as 0.1 mm from one time point to the next) can also disrupt results, especially in case of correlation based connectivity analysis methods, where such small but temporally concordant noise leads to spurious increase in connectivity strength [2], [9], [16]. Nuisance signal regression approaches aim to eliminate the signal components of non-neuronal origin from the raw BOLD data utilizing linear regression. These confounder signals can be defined by dedicated physiological monitoring devices during the scan, calculated from motion parameters extracted during spatial realignment based motion correction or derived directly form the data itself, using a “noise ROI” [6], [7], [17].

Temporal filtering is also a crucial step in the reduction of fMRI brain connectivity artifacts, including motion confounds. Most connectivity studies apply a band-pass filter with a high-pass cutoff of 0.008–0.01 Hz and a low-pass threshold of 0.08–0.1 Hz [18]. While there is evidence that resting-state networks are present at a relatively broad band in the frequency spectra [19], slight modifications in the frequency band have been suggested [3].

Some of these techniques can effectively reduce not only motion-related effects, but also physiological noise (e.g. cardiac or respiratory confounds) or hardware drifts and instabilities. However, recent studies [3], [8], [20] report that clear artifacts remain in the data even after such regression and filtering approaches, and that these artifacts have systematic effects upon resting-state functional MRI connectivity (rs-fcMRI) patterns.

When performing group comparisons in functional connectivity studies, one can account for these motion-related residual artifacts during the second-level analysis by inclusion of motion-related, subject-specific covariates into the population-level model. A common choice is to include a measure of the average patient movement [8]–[10]. Alternatively, the value of global voxel-to-voxel correlation (GCOR) can be utilized as confounder, as in [20], although the latter quantity can also carry valuable neurological information.

Due to in-scanner head rotation, the effect of patient movements on the BOLD signal is not spatially constant in the whole brain; however, this local relationship is poorly understood and is rarely considered in brain connectivity studies. The pattern of voxel-wise motion not only varies among different loci of the same subject, but also among subjects. According to Satterthwaite et al. [9], between-subject differences of motion are stable and hence, in-scanner head motion should be considered as a trait. Thus, the effect of the subject-specific spatio-temporal motion pattern on the BOLD signal could bias group analysis when different groups have different tendencies in their spatio-temporal motion patterns. This is particularly problematic in studies when regional connectivity deficits are associated with a pathological condition, and thus, limits the usability of functional connectivity as a biomarker of disease. These biases in the functional connectivity pattern can lead to invalid conclusions regarding biomedical hypotheses, as denoted by [21] and demonstrated by [20], especially in patholological conditions associated with hyperkinetic patients (epilepsy, attention deficit hyperactiviy disorder, some forms of autism). Group-wise inconsistencies in motion patterns can arise from different patient positioning and multi-center studies are also challenging in this regard.

### Purposes

Here, we hypothesize that at least some of the above-mentioned artifactual effects may originate from the complex voxel-wise spatio-temporal nature of head displacement, and can be modeled more efficiently using this information.

The possibilities for utilizing the voxel-wise nature of in-scanner motion in artifact removal approaches has not been intensively investigated, as yet. As recently reported by two studies [3], [14] and confirmed by our preliminary analysis, including voxel-wise displacement parameters as voxel- or region-wise covariates in the appropriate nuisance signal regression model does not significantly improve motion artifact removal, compared to the usual technique, the regression of spatially averaged global displacement parameters. However, Yan et al. in [14] also brings up the possibility that an appropriate correction technique may have greater success in using the rich information encapsulated by voxel-specific indices.

Our study was designed to characterize the impact of voxel-wise head motion artifacts in population-level rs-fcMRI brain connectivity studies and investigate how this local information on displacement can be utilized for artifact removal.

We initially demonstrate the local correspondence between head displacement and the changes in the rs-fcMRI BOLD signal. We then aim to investigate how functional connectivity strength is affected by the deviations in the average regional spatial displacements on the population-level. We propose Regional Displacement Interaction (RDI), a novel modeling approach for second-level brain connectivity analysis, which provides the opportunity to incorporate voxel-wise motion information into the population-level model and to account for corresponding artifactual effects. The effectiveness of this motion artifact reduction technique is evaluated by investigating the variance explained by the proposed confound modeling covariates in the model. The method is than demonstrated in group comparisons of cohorts with differing average voxel-wise displacement patterns. Due to the disagreement [3], [14], [20], [22] about the optimal first-level nuisance signal regression technique, we perform a comparison of prevailing first-level nuisance signal regression approaches and characterize their interference with the proposed method. Finally, to test whether the proposed method preserves group differences of neuronal origin, a comparison of autistic and control groups is performed.

## Materials and Methods

### Image acquisition

Analysis was performed on the resting-state fMRI data of 184 patients obtained from the Autism Brain Imaging Dataset Exchange database [15], [23], [24] (ABIDE). All of the images were acquired at the NYU Langone Medical Center using a 3 Tesla Siemens Magnetom Allegra syngo MR 2004A. A T1-weighted sagittal MP-RAGE structural image was obtained (TE = 3.25 ms, TR = 2530 ms, TI = 1100 ms, flip angle = 7, 256 slices with 1.3×1×1.3 mm voxels). Functional images were obtained using a BOLD contrast sensitive gradient echo echo-planar sequence (TE = 15 ms, flip angle = 90, in-plane resolution = 3×3 mm; volume TR = 2000 ms). Whole-brain coverage for the functional data was obtained using 33 contiguous interleaved 4 mm axial slices. During the resting-state fMRI scan, most participants were asked to relax with their eyes open, while a white cross-hair against a black background was projected on a screen. However, data were also included for some individuals who were asked to keep their eyes closed; and, in a few cases, participants closed their eyes regardless of instructions to keep them open.

The population sample consisted of 79 patients with autism spectrum disorders (7.1–39.1 years) (53 Autistic Disorder, 21 Asperger's Disorder, 5 Pervasive Developmental Disorder-Not Otherwise Specified) and an age and gender-matched group of 105 typical control subjects (6.5–31.8 years).

Data collection for the ABIDE dataset was approved by the institutional review boards of the New York University School of Medicine and New York University. Prior to participation, written informed consent and assent (for participants>18 years) were obtained from all participants and their parents/legal guardians (for participants<18 years). Participants received monetary compensation for completing the study. In this study, the patient data were analyzed anonymously.

### Preprocessing

FMRI time series were co-registered and frame-wise estimation of head displacement was performed using FSL McFlirt [25], [26]. Matrices defining the rigid-body (three translation and three rotation parameter) transformation that fit each frame to the reference frame (at the middle time-point) were saved for further use. The first five volumes of each dataset were discarded from further analysis to allow for T1 equilibration effects. BET was used to remove non-brain areas [27]. The resulting pre-processed fMRI data were nonlinearly co-registered to the brain-extracted anatomical image, and then, spatially standardized to the MNI152 space using the FLIRT and FNIRT utilities [28] of the FSL package, to achieve spatial correspondences for group analysis. Since further processing steps utilized averaged regional time courses, no smoothing was applied on the images.

### Calculation of voxel-wise displacement

**Figure 1:**
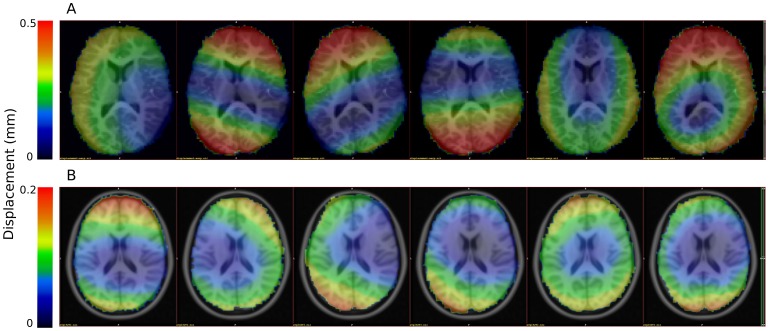
Voxel-wise characteristics of head motion during an fMRI scan. Examples of different patterns of voxel-wise displacement within the time frames of one illustrative subject (A) and temporally averaged voxel-wise displacement of six illustrative subjects (B).

With an in-house-developed utility based on the m3i software library system [29], transformation matrices outputted by McFlirt were converted to world coordinate origin. The respective inverse transformations were applied to each frame of the fMRI time-series and the root mean squared voxel position change in world coordinates was calculated for each voxel of each frame. The first derivate of the resulting local displacement time-series was saved in NIfTI format dynamic images in the same space as the fMRI time-series (see Fig. 1 for demonstrative images), and then realigned to standard space.

### ROI definition

**Figure 2:**
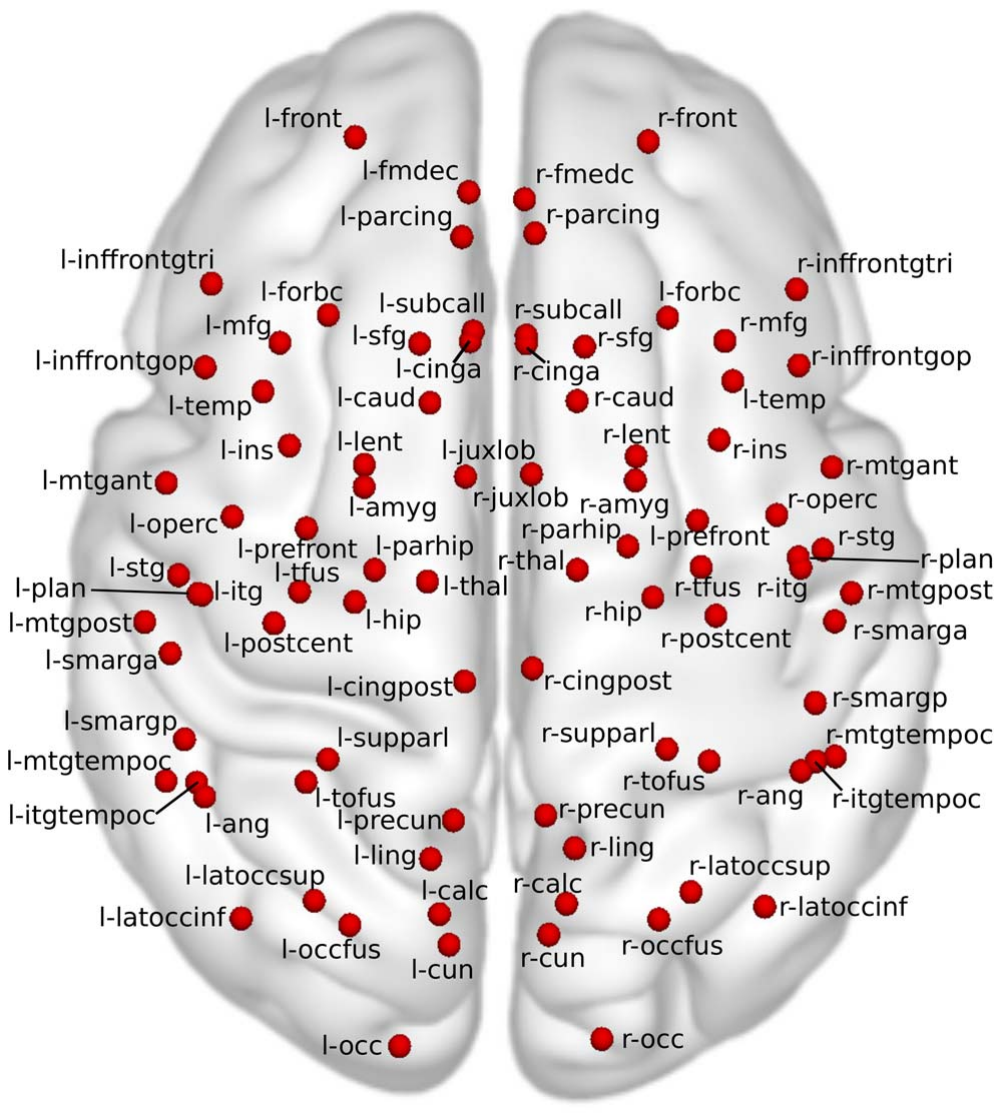
Brain atlas regions. Short names of atlas regions in the glass brain plot used to demonstrate results. Full names and additional information about regions can be seen in Table 1. Red spheres imply the axial projection of the center of mass of brain regions. Note that this plot does not indicate the axial depth of the regions.

In order to improve the signal-to-noise ratio and reduce the amount of data to analyze, all regional timecourses (regional BOLD signal, temporal derivate of its root mean squared variance, regional displacement) and corresponding correlation coefficients presented in this paper were drawn from a set of ROIs (M = 88) that were defined based on the Harvard-Oxford Cortical and Subcortical brain atlases [30]. Probability maps for all regions were accessed and region borders were delineated by retaining voxels with a probability greater than 25%. Voxels associated with multiple regions (in case of overlapping regions) were assigned to the region in which the underlying probability was higher. To avoid very small regions with poor signal-to-noise ratio, ROIs having a volume less than 30 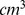 were merged into neighboring ROIs. A complete list of the brain regions and the modifications are summarized in Table 1. Fig. 2 presents the axial projection of brain regions in the glass-brain plot used to demonstrate results.

**Table 1:** Brain atlas regions, left hemisphere.

id	long name	hemisphere	short name	H-O atlas regions merged
1	Left Caudate	left	l-caud	Caudate, Accumbens
2	Cingulate Gyrus anterior division	left	l-cinga	
3	Frontal Medial Cortex	left	l-fmedc	
4	Operculum	left	l-operc	frontal operc, central operc, parietal operc
5	Frontal Orbital Cortex	left	l-forbc	
6	Frontal Pole	left	l-front	
7	Inferior Frontal Gyrus pars opercularis	left	l-inffrontgop	
8	Inferior Frontal Gyrus pars triangularis	left	l-inffrontgtri	
9	Juxtapositional Lobule Cortex	left	l-juxlob	
10	Middle Frontal Gyrus	left	l-mfg	
11	Paracingulate Gyrus	left	l-parcing	
12	Pre-frontal	left	l-prefront	
13	Subcallosal Cortex	left	l-subcall	
14	Superior Frontal Gyrus	left	l-sfg	
15	Insular Cortex	left	l-ins	
16	Cuneal Cortex	left	l-cun	
17	Calcarine Cortex	left	l-calc	intracalcarine, supracalcarine
18	Lateral Occipital Cortex inferior division	left	l-latocinf	
19	Lingual Gyrus	left	l-ling	
20	Occipital Fusiform Gyrus	left	l-occfus	
21	Occipital Pole	left	l-occ	
22	Angular Gyrus	left	l-ang	
23	Cingulate Gyrus posterior division	left	l-cingpost	
24	Lateral Occipital Cortex superior division	left	l-latoccsup	
25	Postcentral Gyrus	left	l-postcent	
26	Precuneous Cortex	left	l-precun	
27	Superior Parietal Lobule	left	l-supparl	
28	Supramarginal Gyrus anterior division	left	l-smarga	
29	Supramarginal Gyrus posterior division	left	l-smargp	
30	Lentiform	left	l-lent	putamen, pallidum
31	Superior Temporal Gyrus	left	l-stg	sup.temp.g. post; sup.temp.g. ant; Heschl gyrus
32	Inferior Temporal Gyrus	left	l-itg	inf.temp.g. post; inf.temp.g. ant
33	Inferior Temporal Gyrus temporooccipital part	left	l-itgtempoc	
34	Amygdala	left	l-amyg	
35	Hippocampus	left	l-hip	
36	Middle Temporal Gyrus anterior division	left	l-mtgant	
37	Middle Temporal Gyrus posterior division	left	l-mtgpost	
38	Middle Temporal Gyrus temporooccipital part	left	l-mtgtempoc	
39	Parahippocampal Gyrus	left	l-parhipc	Parahippocampal Gyrus post.; Parahippocampal Gyrus ant.
40	Planum	left	l-plan	Planum Temporale, planum polare
41	Temporal Fusiform Cortex	left	l-tfus	fusiform ant, fusiform post
42	Temporal Occipital Fusiform Cortex	left	l-tofus	
43	Temporal Pole	left	l-temp	
44	Left Thalamus	left	l-thal	

Lateralization, and long and short names of brain atlas-based ROIs used for estimating regional BOLD and motion related measures. The sources of brain regions are the Harvard-Oxford Cortical and Subcortical brain atlases [30]. Probability maps for all regions were accessed and region borders were delineated by kretaining voxels with a probability greater than 25%. Voxels associated with multiple regions (in case of overlapping regions) were assigned to the region in which the underlying probability was higher. ROIs having a volume less than 30 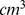 were merged into neighboring ROIs, as indicated by column five. The table lists only regions in the left hemisphere. The naming conventions and the region merging procedure was analogous for their contra-lateral pairs.

### Calculation of regional and frame-wise displacement

We defined two metrics of displacement: regional and frame-wise displacement (RD and FD). RD time-courses were calculated as averaged voxel-wise displacements over ROIs, while FD is the analogous measurement for the entire brain. This method of calculating FD and RD is analogous to the parameter 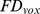 described in [14].

### Quantification of global and regional BOLD intensity change

While DVARS (D referring to temporal derivative of timecourses and VARS referring to RMS variance over voxels) [31] indexes the rate of change of BOLD signal across the entire brain at each time-point of the data, Regional DVARS (RDVARS) shows the same rate for each ROI:
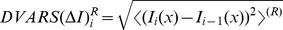
(1)where 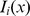 is the image intensity at locus 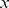 on frame 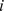, angle brackets 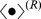 denote the spatial average over all voxels 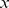 within ROI 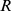 (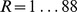) and 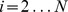 where 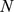 is the number of time-frames. In order to effectively relate RDVARS to RD, RDVARS was calculated on the timeseries following re-alignment, but prior to confound regression and filtering.

### Investigating the effect of regional displacement on DVARS

We reproduced results showing global motion-related BOLD changes [2], [14], [16] by computing the correlation coefficient between FD and DVARS. Then, to distinguish global from local effects, we defined two measures, residual RD and residual RDVARS (denoted with 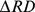 and 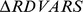), as follows:
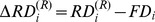
(2)and
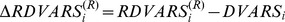
(3)where 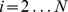, 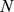 is the number of time-frames and 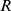 (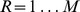) identifies the region. After computing these measures for every subject and every ROI, we computed their correlation coefficient and investigated whether it depends on the degree of the global motion-BOLD relationship among subjects.

### Functional connectivity processing and graph formation

For rs-fcMRI analysis, additional preprocessing steps were utilized on regional BOLD timecourses to reduce spurious variance that was unlikely to reflect neural activity. These steps included: (i) multiple regression of nuisance variables; and (ii) a temporal band-pass filter on residual data using a standard fourth-order Butterworth band-pass filter, retaining frequencies between 0.01 and 0.1 Hz.

The detailed data processing steps involved in the following strategies are discussed in the relevant works. Here, we only summarize the protocols based on basic criteria, such as the sources of nuisance signal, the number of such signal time-courses and whether global signal regression was performed.

1. NOREG: No nuisance regression,
2. WMCSF: average BOLD signals of eroded white matter and cerebrospinal fluid ROI-s, segmented using FAST [27],
3. GSREG: regression of whole-brain global signal as a covariate,
4. COMPCOR: Nuisance regression of five principal components of a noise ROI, defined based on the temporal signal-to-noise ratio, as proposed in [7],
5. NOREG+M6: NOREG + six motion parameters,
6. WMCSF+M6: WM+CSF regression + six motion parameters,
7. GSREG+M6: GSREG + six motion parameters,
8. COMPCOR+M6: COMPCOR + six motion parameters,
9. SAT36: A 36-parameter nuisance regression model proposed in [3] (incorporating global signal regression).

To ensure that neighboring regional BOLD time courses do not show spurious increase in connectivity strength, no smoothing was applied. Connectivity strength between pairs of preprocessed regional time series were calculated as the Fisher-Z transformed Pearson product moment (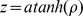), and ordered into 88×88 correlation matrices for each subject (hitherto referred to as 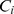 for subject 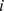) and for each nuisance regression technique, resulting in a total of 184*9 = 1656 matrices.

### Group formation

**Figure 3:**
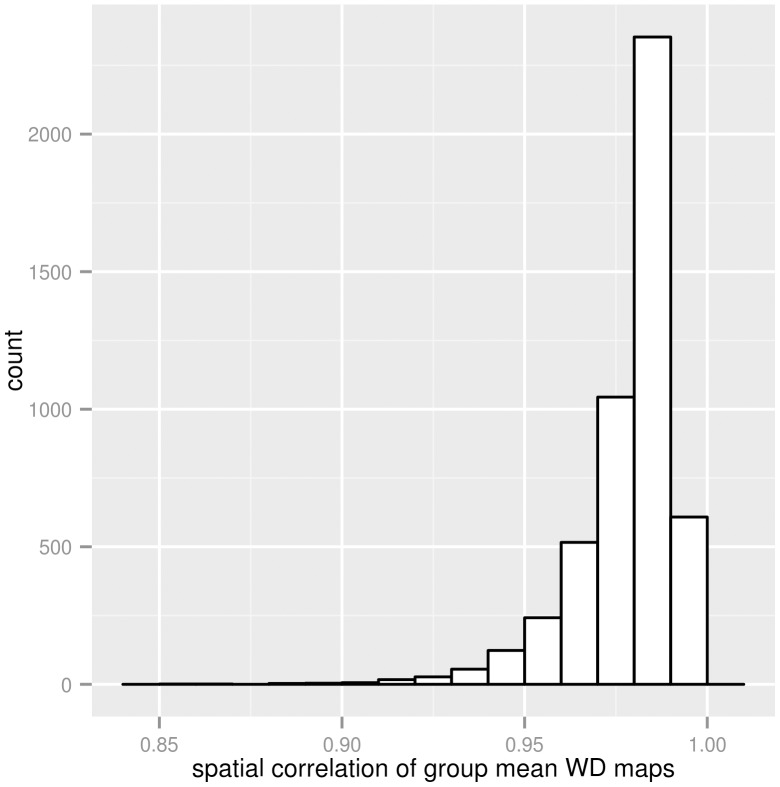
Histogram of group averaged voxel-wise displacement correlations based on 5000 random permutations. Temporally averaged standard-space voxel-wise displacement maps were averaged across the subjects of two randomly assigned groups. Spatial Pearson correlation was calculated between these group-mean voxel-wise displacement maps 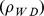). The histogram of this inter-group voxel-wise displacement correlation was computed based on 5000 random group formulation. Group-pairs with extrem inter-group differences in voxel-wise displacement were chosen for further analysis.

One subject with extreme in-scanner motion (average FD greater than 0.7 mm) was excluded from further analysis. The remaining 183 subjects were arranged into various groups:

- Autistic and normal control groups. Based on the clinical neuropsychological diagnostic tests for autism, as detailed in the original study description of the ABIDE dataset [15]. A group of 49 autism subjects and a neurotypical control group (n = 105) were defined.
- Groups of healthy control patents with different average voxel-wise displacement patterns, based on the group mean voxel-wise displacement maps. The healthy control group was divided into two sub-groups randomly. Temporally averaged standard-space voxel-wise displacement maps were averaged across subjects of both sub-groups separately and the spatial Pearson correlation coefficients for the group-mean voxel-wise displacement maps were calculated between the sub-groups (hitherto referred to as 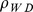). This random group formation was repeated 5000 times to generate groups-pairs with different between-group voxel-wise displacement correlation. The histogram of observed 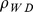 values can be seen in Fig. 3. Group-pairs were chosen so that the corresponding 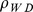 values are relatively low, meaning that the subjects in one sub-group tend to have different voxel-vise displacement patterns than in the other. We chose eight pairs of groups with 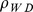 coefficients between 0.89–0.95 (Table 2). 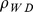 was also computed for the autism-related group-pair.

**Table 2:** Voxel-vise displacement-dependent groups.

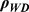	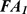	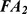	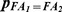	max 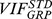	max 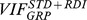
0.89	0.06 (±0.03)	0.08 (±0.04)	0.011	1.11	1.2
0.90	0.07 (±0.04)	0.07 (±0.03)	0.46	1.24	1.09
0.91	0,07 (±0.03)	0.07 (±0.04)	0.99	1.36	1.00
0.92	0.07 (±0.03)	0.07 (±0.04)	0.11	1.37	1.08
0.93	0.07 (±0.03)	0.08 (±0.04)	0.12	1.41	1.07
0.94	0.07 (±0.02)	0.07 (±0.04)	0.78	1.38	1.1
0.95	0.07 (±0.03)	0.07 (±0.04)	0.67	1.23	1.02
0.96	0.07 (±0.03)	0.07 (±0.03)	0.82	1.21	1.01

Formation of voxel-wise displacement related groups. N = 105 healthy control patients were divided into group-pairs randomly, 5000 times. Eight pairs of groups were chosen so that correlation between the group-mean voxel-wise displacement maps (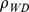) were 0.89, 0.9, 0.91, 0.92, 0.93, 0.94, 0.95 and 0.96. 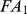 and 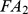 denotes the mean (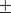 standard deviation) FD of the group-pairs (in mm) and 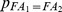 denotes the probability that the groups are identical regarding FD (obtained using permutation test). Max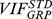 and max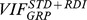 denotes the obtained maximal variance inflation factor (VIF) (throughout all connections) corresponding to the grouping factor, in models STD and STD+RDI, respectively. None of the groups introduce multicollinearity in the models. (however there is a slight difference between the FDs of the group-pairs with a spatial voxel-wise displacement correlation of 0.98).

### Group comparisons

Group comparisons were performed by fitting Generalized Linear Models (GLM) [32] and arranging statistical parameters into differential statistical parametric networks (SPNs) [33]. We investigated the effect of the grouping variable on functional connectivity strength. Additional covariates that might significantly influence functional connectivity, and thus, disturb comparison, were also included in the models. These are phenotypic covariates describing age, full-scale Wechsler Abbreviated Scale of Intelligence (full IQ), gender, and subject-specific mean FD. Connectivity strength is therefore modeled as:
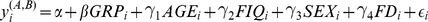
(4)where 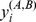 is the measured connectivity strength between regions 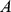 and 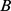 for subject 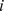 (element at the 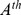 row and 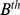 column of the 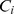 matrix), 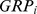 is the dummy variable coding groups to compare, and 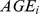, 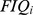, 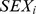 and 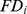 are the aforementioned subject-specific confounder variables, 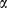, 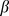 and 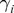
s are the coefficients to estimate, and 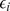 is the the 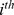 independent identically distributed normal error. Models were fitted utilizing an iteratively reweighted least squares (IWLS) algorithm. T-scores and p-values of the effect of interest were obtained by dividing the 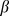 coefficient of interest by the estimated standard error.

#### Regional Displacement Interaction – RDI

According to our hypothesis, differences in the mean RDs of region-pairs 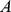 and 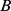 may have an important effect on the population-level distribution of correlation coefficients corresponding to the given connection. This can be tested by adding new terms to the linear model: the temporally averaged *RD*s of regions 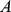 and 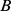 and, furthermore, the interaction term between these two covariates. Since *RD*s are strongly correlated with each other and with the global FD, we introduced 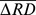 which is an alternative to the average RD, but with FD subtracted, in order to avoid multicollinearity in the model. Accordingly, 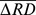 can be defined as follows:
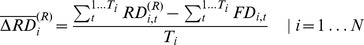
(5)or using the terminology of equation (2):
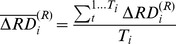
(6)where 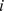 denotes the subject, 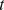 the time frame, 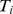 the number of time frames in the fMRI time series of subject 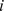 (after exclusion of first five volumes).

Thus, in case of a GLM model for the connection between regions A and B, equation (4) extends to:
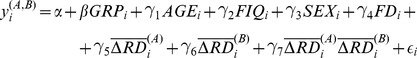
(7)


Henceforward, we refer to the model specified in equation (4) as a standard (STD) model and, to the terms 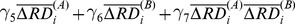 as *regional displacement interaction* (RDI) and, to the model defined by equation (7) as an STD+RDI model.

#### Characterization of the RDI effect

To investigate the RDI interaction effect on connectivity strength we utilized the following models:
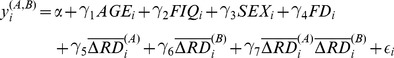
(8)and
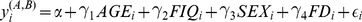
(9)which are alternative versions of models (7) and (4), respectively, with GRP variable excluded. Since models (8) and (9) are nested, we can compare the reduction in deviance to residuals utilizing an F-test under the null hypothesis that none of the additional RDI covariates in the STD+RDI model is related to the measured connectivity strength. The resulting statistical parameters for each connection were ordered into nine differential SPNs for each nuisance regression model, showing connections significantly related to RDI. Model (8) was also used to demonstrate RDI in case of a single, representative connection.

We furthermore fitted a model defined as:
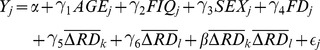
(10)where 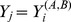 so that 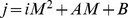 and 
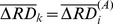
 so that 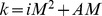 and 
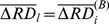
 so that 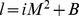, or, in other words, the dependent and independent variables of model (8) are concatenated following the A and B (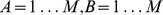) atlas regions. In contrast to the previously introduced models, here, we utilize one model for all connections of all subjects. The effect of interest is the RDI interaction term. This model was used to analyze the overall nature of the interaction term.

To avoid the disturbing effect of autistic differences when characterizing the interaction term, only the healthy control population was involved when applying models defined by Eqs. (8), (9) and (10).

#### Voxel-wise motion-related group comparison

Differences between low and high motion groups were investigated by two GLM models for every connection: STD (Eq. (4)) and STD+RDI (Eq. (7)) models were applied where GRP was a dummy variable that defined the motion-related groups as listed in Table 2. Results were ordered into 8*9*2 t-score SPNs, summarizing motion-related group differences (eight pairs of motion-related groups, nine first-level nuisance regression methods, and two second-level regression models [STD and STD+RDI]). Since, in these comparisons, the variable of interest (grouping factor) and the motion-related covariates (FD and the RDI covariates) are potentially related, we computed the variance inflation factor (VIF) [34] for each model. VIF values suggest that these modeling approaches are free from multicollinearity issues. (Maximal observed VIF values for the variable of interest are reported in Table 2.)

#### Autistic-control comparison

We tested the proposed RDI second-level interaction covariate set by comparisons of autistic and control groups defined by phenotypic information that was provided with the Autism Brain Imaging Dataset Exchange database. Results were arranged into 9*2 SPNs for nine nuisance regression methods and two second-level regression methods (STD and STD+RDI).

### Computations and network visualization

Computations in this study, when not specified otherwise, were performed using R statistical programming language [35], using the packages “glm” [36], “fdrtool” [37], [38], “HH” [39] and “visreg” [40]. Differential SPNs were thresholded and visualized with the in-house developed software BrainCON (www.minipetct.com/braincon) [29].

## Results and Discussion

### fMRI motion artifacts have spatial predisposition

As noted by Power et al. [2], [41], the effect of motion appears to scale with the amplitude of the displacement over the whole brain: frames with greater amplitude displacements are associated with a greater change in BOLD signal. As a first step, we reproduced these results by observing a 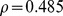 (p<0.000001) correlation between frame-wise displacement FD and DVARS.

**Figure 4:**
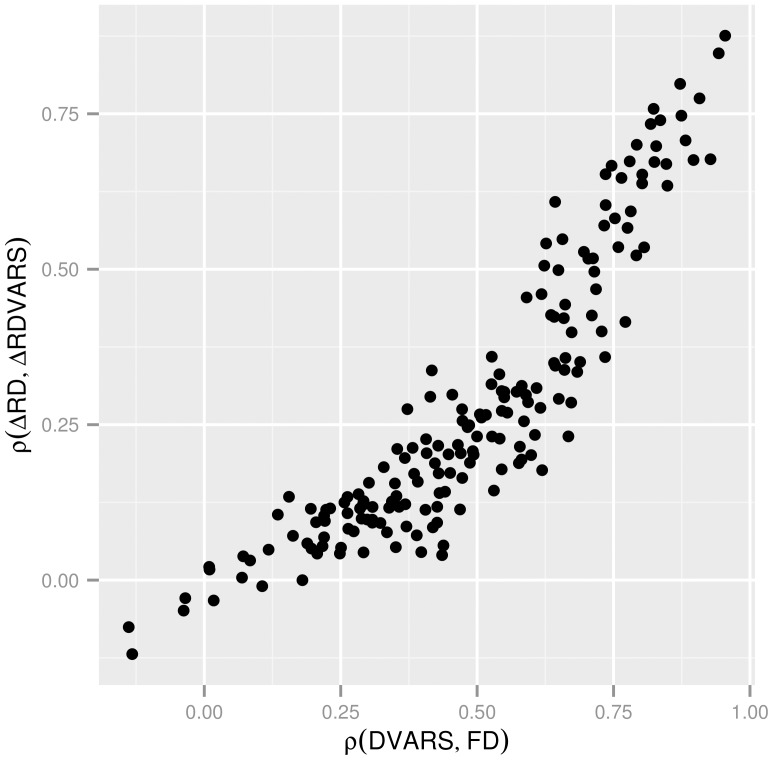
Regional motion-BOLD relationship scales with the global motion-BOLD relationship. Within-subject correlation of 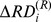 and 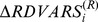 (horizontal axis) plotted against the correlation of FD and DVARS (vertical axis) for all brain regions and time frames of N = 183 subjects. The regional effect of motion on BOLD seems to scale with the global relationship which is correlated (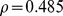 (p<0.000001) with the mean FD.

Our results also show that this effect is not spatially constant. Although as also reported in [3], RD time courses a show high correlation in the entire brain of one subject, if the global effect is subtracted from the regional measures (FD from RD and DVRAS from RDVARS, respectively) the resulting residual measures 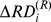 and 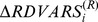 (Eqs. (2) and (3)) still show a significant correlation of 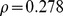 (p<0.0001). In addition, as presented in Fig. 4, the correlation of these residual regional measures increases with the global motion-BOLD relationship throughout subjects.

In-scanner head motion consists not only of translations. Rotational components make the displacement diverse in distinct locations: it becomes greater when moving further from the center of rotations. A plausible explanation of the reported regional relationship is that this complex spatio-temporal pattern of displacement implicitly affects local BOLD signal changes.

These results can also yield a potential explanation of the phenomenon that motion tends to increase connectivity for locally adjacent nodes, but reduces connectivity between distant nodes [2], [8], [9], [14], [16]: neighboring regions having more similar RD will share more similar motion artifacts than regions being far from each other and this effect biases the distance dependence of connectivity strength.

However, we should point out that all the discussed voxel-wise and regional displacement measures are only “apparent displacements”: their estimated values may have been affected by phenomena other than head motion, including physiological noise, magnetic field inhomogeneities, instrumental instabilities, as well as BOLD activity of neuronal origin. This effect should be more pronounced for regional displacements than for the frame-wise displacements. Furthermore, the computation of RD implicitly include integrating of motion effects within each fMRI volume. Rapid head movements occurring on time scale shorter than the fMRI repetition time TR may affect different slices within an fMRI volume differently [42]. Effects of such rapid movements cause slice-specific image distortions that cannot be accurately taken into account by the volume realignment-based procedure, but can still affect fMRI functional connectivity results. Averaging over all time frames and within time frames is a simplification in modeling the spatial predisposition of head motion. However, as suggested by the significant correlation between 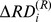 and 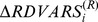, this simplification seems to be reasonable. Nevertheless, sub-TR frequency components of in-scanner motion deserve more attention and their voxel-wise effect should be investigated in more detail in future publications.

### The interaction of regional displacements affects measured connectivity strength

Evidences of a spatially non-constant motion artifact in brain connectivity analysis, like an increase in short-range and a decrease in long-range connectivity, or the special pattern of related changes in connectivity strength reported in [2], [8], [9], [14], [16], suggest that the reported local relationship between motion and BOLD signal changes should be considered when performing correction techniques. This is especially true for correlation-based functional connectivity analysis, where the similarity of two regional BOLD time courses can be increasingly affected by these small but systematic variations.

However, including voxel-wise motion parameters in nuisance signal regression does not seem to be efficient (as reported by [3], [14] and also found in our preliminary analysis). Yan et al. [14] used voxel-wise displacement as a reference to evaluate the differential region-specific impact of motion on the BOLD signal. Although these authors presented significant correlations with a spatial pattern similar to that previously reported, it is still not clear whether those patterns can be explained only by locally differential BOLD answers to a global motion effect, or, alternatively, by a real local relationship with the spatio-temporal motion pattern.

**Figure 5:**
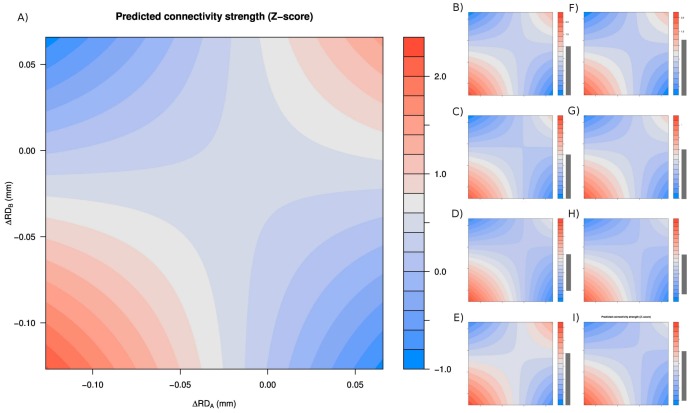
Filled contour plots visualizing the Regional Displacement Interaction (RDI) effect: how the predicted connectivity strength (color-coded) changes depending on the simultaneously varying values of 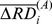 and 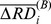, in case of no nuisance regression (A∶NOREG) and all investigated first-level nuisance regression methods, i.e., NOREG+M6 (E), WMSCF (B), COMPCORR (C), GSREG (D), WMSCF+M6 (F), COMPCORR+M6 (G), GSREG+M6 (H), and SAT36 (I). Vertical and horizontal axes of plots B-I are the same as those of plot A. Gray bars next to the legends indicate the (−1,1) interval to ease interpretation of color-coded Z-score values.

To investigate this question, we defined RDI, a set of second-level regression covariates that models the interaction effect between the temporally averaged regional displacements of the regions involved in the connection. In regression analysis, an interaction effect is said to exist when the effect of the focal independent variable on the dependent variable differs depending on the value of a third variable [43], called the moderator variable. (Statistically, the choice of which of the two independent variables should be the moderator variable is unimportant.) The proposed RDI interaction term in the second-level GLM model defined by Eq. (10) was found to be significant (p<0.000001) for all first-level nuisance regression methods), which means that the effect of the average RD of one region on the dependent variable (connectivity strength) changes when the average RD of the other region changes. The effect is visualized in Fig. 5 by the filled contour plots. The predicted connectivity strength changes depending on the simultaneously varying values of 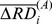 and 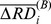, in case of all investigated first-level nuisance regression methods. These results imply that throughout the population, connectivity strength between two brain regions tends to increase if the average RD of the regions is similar (eg. both are larger or smaller than the average FD) and tends to decrease otherwise.

**Figure 6:**
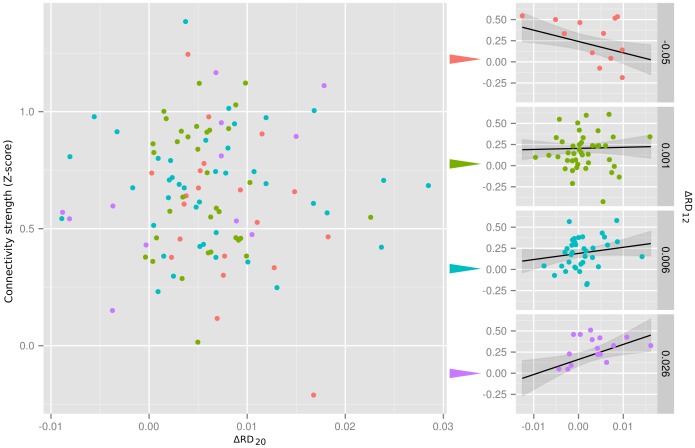
RDI effect in case of a demonstrative connection: occipital fusiform gyrus (A = 20) - prefrontal gyrus (B = 12). On the left, the partial residuals for 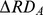 from the model defined by Eq. (8) (dependent variable: connectivity data of the healthy control population (N = 105), without nuisance signal regression (NOREG)) are plotted against 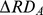. Although the model reveals no significant relationship (t = −1.46,p = 0.15) between connectivity strength and 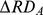, the 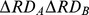 interaction effect is significant (t = 3.31, p = 0.0013), implying that the effect of 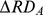 on connectivity strength differs depending on the value of 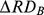. This is demonstrated by dividing the data into four groups based on the value of 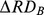 (color-coded on the left plot) and visualizing the corresponding cross-sectional CCPR (component and component-plus-residual) plots (on the left). Mean value of 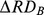 corresponding to the cross-section is indicated. Partial residuals are plotted with colored dots corresponding to the cross-section group. The corresponding regression line estimated from the full model fit and the corresponding 95% confidence interval is displayed in black and gray, respectively. The horizontal and vertical axes of the cross-sectional CCPR plots are the same as those of the partial residual plot on the left. Cross-sectional CCPR plots imply that in each group the relationship between 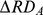 and partial residual connectivity strength is significant but the regression lines have different slopes, which makes this latent relationship not observable without accounting for the interaction of the regional displacement covariates.

This effect is demonstrated for a representative connection (occipital fusiform gyrus - prefrontal gyrus) in Fig. 6. While the presented partial residual plot reveals no significant relationship between connectivity strength and 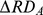, the 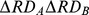 interaction effect is significant implying that the effect of 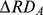 on connectivity strength differs depending on the value of 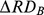. This is demonstrated by dividing the data into four groups based on the value of 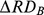 and visualizing the corresponding cross-sectional CCPR (component and component-plus-residual) plots, which reveal that in each group the relationship between 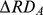 and partial residual connectivity strength is significant in all cases but the regression lines have different slopes. Thus, this latent relationship is not observable without accounting for the interaction of the regional displacement covariates.

**Figure 7:**
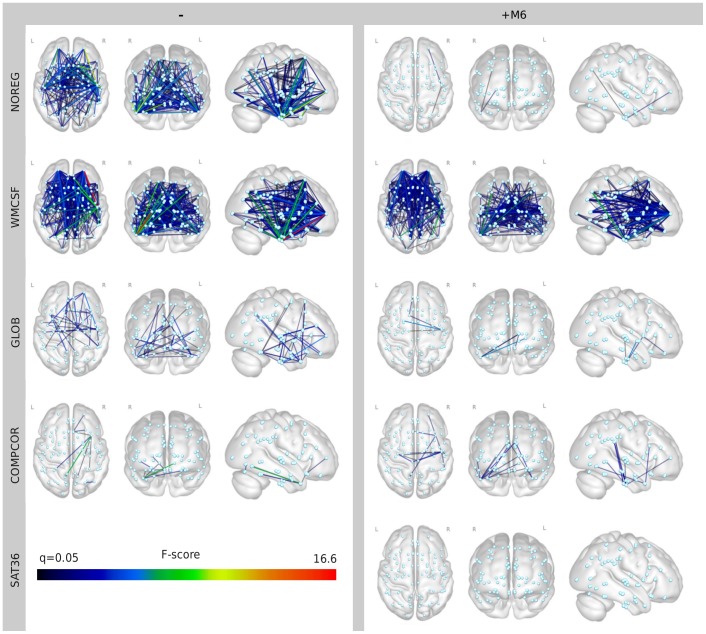
Network pattern of connections where utilizing RDI significantly improves second-level modeling. Statistical parametric networks presenting the model comparison performed by F-tests between the STD and STD+RDI models (Eq. (9) and (8)). Connections are only visualized, when the STD+RDI model explains significantly more variance than the STD model (the null hypothesis of the F-test can be rejected) with a false discovery rate of q<0.05. The proposed STD+RDI method proves to be most efficient with the nuisance signal regression methods NOREG, WMCSF, WMCSF+M6, GSREG, and COMPCOR+M6, and seems to demonstrate no significant improvement in case of SAT36, after correction for multiple comparison.

To characterize how this phenomenon is related to the spatial patterns of measured functional connectivity and to what extent it is present in case of the applied first-level nuisance signal regression techniques, we performed a comparison of the STD (Eq. (8)) and STD+RDI (Eq. (9)) models. The model comparison was realized by F-tests between the models and resulted in the SPNs shown in Fig. 7. In this figure, connections are visualized, where the STD+RDI model explains significantly more variance than the STD model (the null hypothesis of the F-test can be rejected) with a false discovery rate of q<0.05. The proposed method, RDI proved to be the most efficient with nuisance signal regression methods NOREG, WMCSF, WMCSF+M6, GSREG, and COMPCOR+M6. The explanatory power added by RDI is most pronounced typically in case of middle- and long-range connections of the temporal poles.

### The interaction of regional displacements may bias functional connectivity group comparisons

The correlation coefficient of two regional BOLD time courses can be sensitive to the regional displacement time course (RD) of both regions. Even if the motion-related artifactual component is small, it still can significantly affect correlations, depending on the degree to which it is shared between the time courses.

This phenomenon becomes even more problematic on the population-level. Satterthwaite et al. [9] reported that between-subject differences in head motion are stable: that subjects who tend to move on one occasion tend to move on another occasion. This means that analyses of functional connectivity needs to consider the possibility that *certain aspects of head motion behave as a trait*. Accordingly, even if the above-mentioned effect is otherwise small, it can disturb group comparisons and lead to erroneous conclusions, since it is of non-neural origin.

This assumption can be admitted easily by considering two patient cohorts where region A and B have similar RD within each subject of one group (e.g., due to relatively smaller rotations), and different RD in the subjects of the other (e.g., more prevalent rotations with a center to which A and B are located asymmetrically). The significant RDI interaction effect means that the corresponding correlation coefficient will be biased, and tends to be larger in the first group, even in the absence of a real functional difference. However, it is still not clear how different grouping conditions interfere with the tendency of motion patterns.

One can hypothesize that the spatio-temporal pattern of motion can be significantly different between groups that were defined by a factor in relation to motion. This could be the case in group comparisons in several physiological and pathological conditions co-occurring with hypo- or hyperkinetic signs.

Our results show that the spatio-temporal pattern of head motion biases measured connectivity strength on the population level, and, practically speaking, *the proposed second-level covariates (RDI) can be utilized as a method to incorporate these individual regional differences of in-scanner head motion into the model, and thus, reduce artifactual variance in the data*.

### Including RDI as a covariate in second-level regression efficiently reduces group differences caused by differences in voxel-wise motion

To demonstrate the reported confounding effect of voxel-wise motion on population-level analysis, we performed eight group comparisons (See Table 2) where the groups to compare have different average voxel-wise displacement patterns.

**Figure 8:**
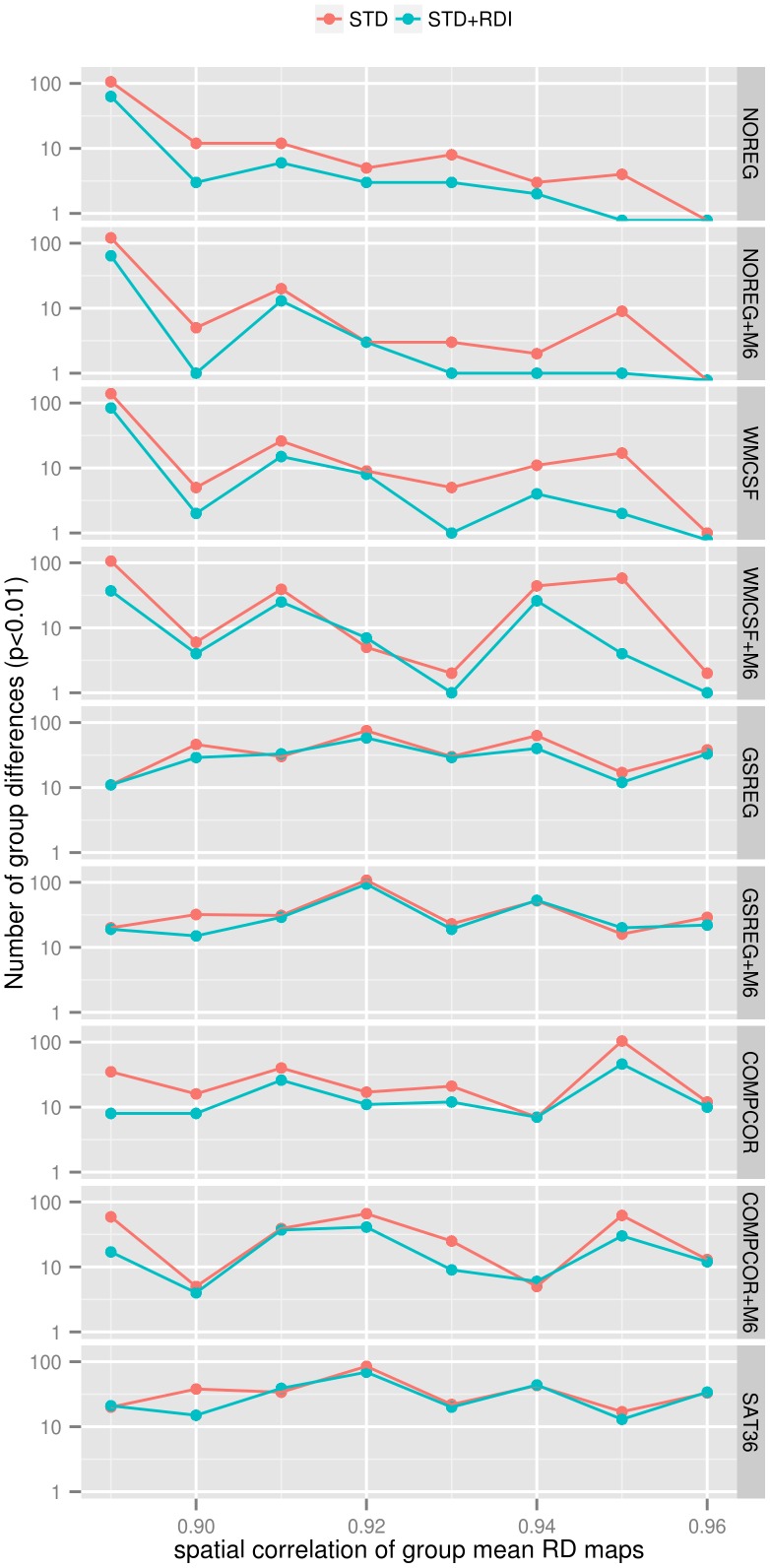
The effect of RDI on motion-related group differences. The number of significantly (p<0.01) differing connections is plotted against the spatial correlation coefficient of group-mean voxel-wise displacement maps for RD-based group-pairs for each nuisance signal regression method. The number of significant group differences is plotted on logarithmic axis. Improvement in the reduction of motion-related group differences was most pronounced for the NOREG, WMCSF and COMPCOR methods. Nuisance regression methods incorporating GSREG seem not to be sensible for differences in group-mean voxel-wise displacement patterns however, they show relatively high number of group differences by all group comparisons.

Results are summarized in Table 3. In Fig. 8, the number of significantly (p<0.01) differing connections is plotted against the group-defining mean FD threshold for each nuisance signal regression method. Results show that, for less extensive nuisance regression methods (NOREG, NOREG+M6, WMCSF, WMCSF+M6), when the voxel-wise displacement maps between groups are more different (lower between-group mean voxel-wise displacement map correlations), more group differences appear. These findings seem to confirm that group comparisons may be biased when groups show different tendencies in voxel-wise motion. Results, in conjunction with the results of the F-test based model comparisons Fig. 7, also show that *the inclusion of RDI covariates seems to decrease these artifactual group differences, especially by moderate nuisance regression methods*.

**Table 3:** Motion-related group differences.

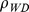	0.89	0.90	0.91	0.92	0.93	0.94	0.95	0.96
NOREG	106 (1)	12	12	5	8	3	4	0
NOREG+RDI	63	3	6	3	3	2	0	0
WMCSF	139 (2)	5	26	9	5	11	17	1
WMCSF+RDI	84	2	15	8	1	4	2	0
GSREG	11	46	30	75 (1)	30	63	17	38
GSREG+RDI	11	29	33	58	29	40	12	33
COMPCOR	35	16	40	17	21	7	104 (2)	12
COMPCOR+RDI	8	8	26	11	12	7	46	10
NOREG+M6	121	5	20	3	3	2	9	0
NOREG+M6+RDI	64	1	13	3	1	1	1	0
WMCSF+M6	106	6	39	5	2	44	58	2
WMCSF+M6+RDI	37	4	25	7	1	26	4	1
GSREG+M6	20	32	31	108 (1)	23	52	16	29
GSREG+M6+RDI	19	15	29	94	19	53	20	22
COMPCOR+M6	59	5	39	66	25	5	62 (1)	13
COMPCOR+M6+RDI	17	4	37	41	9	6	30	12
SAT36	20	38	34	85	22	43	17	33
SAT36+RDI	21	15	39	69	20	44	13	34

Number of connections significantly (p<0.01) differing between groups. Voxel-wise displacement-related group comparisons corresponding to given 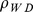 are presented in columns. Rows correspond to the nine nuisance regression methods and their +RDI variants. Number of differences surviving the q<0.05 false discovery rate criterion (if any) is indicated in parentheses.

The change in the corresponding connectivity pattern is visualized in Fig. 8 for the nuisance signal regression methods NOREG, COMPCOR, and GSREG, and for each motion-related group comparison defined by 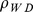. Results with the STD and STD+RDI second-level regression models are presented in the upper and lower rows on each panel, respectively. Group differences with probability (p> = 0.01) are not visualized.

As predicted by the F-test-based model comparisons (Fig 7), the reduction of false group differences caused by voxel-wise motion most markedly improved in the NOREG, WMCSF and COMPCOR methods. Regressing out six motion parameters did not seem to be highly efficient but may be beneficial when no other nuisance signal regression covariate is applied (NOREG+M6).

However, the small number of differences surviving the q<0.05 false discovery rate criterion implies that - when utilizing a proper second-level model - all the investigated methods are able to reduce motion-related group comparison artifacts to a decent extent. With the RDI correction, no FDR significant group differences remained in any of the comparison cases.

We note, that, in contrast to most prior studies, here, we performed more than a single pair of group comparisons, thus avoiding that results reflect only the random effects of the grouping condition.

### Including RDI as a covariate in second-level regression preserves autism-related group differences

To test the efficiency of the proposed correction method, we performed group comparisons where both motion-related artifacts [20], [21] and real neuronal differences [10], [15], [44] were expected to be present. We compared the functional networks of autistic and control patients.

The correlation between the group-mean voxel-wise correlation map 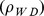 between the autistic and the normal control groups was found to be 0.98. It suggests that this grouping condition should be only slightly biased by the effect of regional displacement on the measured connectivity strength. Thus, as predicted by voxel-wise displacement pattern-related group comparisons, utilizing the RDI correction method should introduce only minor changes in the differential connectivity patterns.

**Figure 9:**
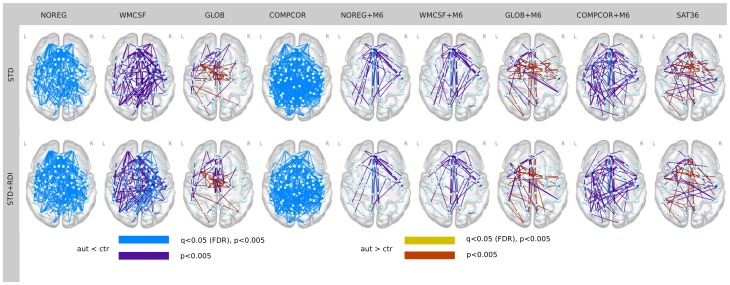
Autism related group comparisons. Autism related group differences for the investigated correction strategies. Colors denote significance levels as detailed in the legend.

Results are presented in Fig. 9 and Tables 4 and 5.

**Table 4:** Autism-related group differences (without nuisance signal regression of six motion parameters).

Connection	NOREG	+RDI	WMCSF	+RDI	GSREG	+RDI	COMPCOR	+RDI
l-cun - l-stg	−3.73 ***	−3.52 ***	−2.79 *	−2.56	−2.67 *	−2.19	−5.03 ***	−4.57 ***
l-occfus - r-tfus	−3.64 ***	−3.98 ***	−3.88 **	−4.03 ***	−3.26 *	−3.17 *	−4.88 ***	−4.62 ***
l-fmedc - r-parcing	−4.30 ***	−4.11 ***	−4.16 **	−4.06 ***	−3.02 *	−3.09 *	−4.86 ***	−4.77 ***
l-fmedc - r-cingpost	−4.54 ***	−4.23 ***	−3.94 **	−3.86 ***	−1.92	−1.94	−4.83 ***	−4.55 ***
r-mfg - r-parhipc	−3.14 ***	−3.63 ***	−4.54 ***	−4.67 ***	−3.70 **	−3.85 **	−3.55 ***	−3.47 ***
l-smargp - l-mtgant	−3.38 ***	−3.54 ***	−2.66 *	−2.79 *	−2.48	−2.54	−4.65 ***	−4.49 ***
r-tfus - r-tofus	−3.35 ***	−4.13 ***	−2.92 *	−3.48 ***	−1.57	−1.75	−4.43 ***	−4.59 ***
l-fmedc - l-cingpost	−4.30 ***	−3.97 ***	−3.52 **	−3.40 ***	−1.83	−1.84	−4.19 ***	−3.95 ***
l-hip - l-thal	−0.28	−0.06	2.2	2.03	4.53 ***	4.21 **	−0.15	0.45
l-sfg - l-mtgant	−3.71 ***	−3.97 ***	−2.84 *	−3.00 *	−3.00 *	−2.97 *	−4.49 ***	−4.30 ***
l-fmedc - r-front	−4.49 ***	−3.98 ***	−4.03 **	−3.61 ***	−2.78 *	−2.45	−4.00 ***	−3.63 ***
l-cun - l-plan	−3.24 ***	−2.96 ***	−2.27	−2.01	−1.72	−1.18	−4.47 ***	−3.96 ***
l-stg - r-cun	−3.19 ***	−2.97 ***	−2.47	−2.24	−2.82 *	−2.45	−4.46 ***	−4.02 ***
l-temp - l-thal	0.28	0.48	2.03	2.16	4.45 ***	4.07 **	0.02	0.13
r-parhipc - r-hip	0.99	0.52	2.33	2.17	4.44 ***	4.29 **	0.34	0.29
l-mtgant - r-sfg	−3.33 ***	−3.50 ***	−2.82 *	−2.89 *	−1.85	−1.73	−4.44 ***	−4.25 ***
r-fmedc - r-precun	−4.43 ***	−4.35 ***	−3.12 *	−3.10 *	−0.89	−1.03	−3.55 ***	−3.67 ***
l-fmedc - l-precun	−4.02 ***	−3.83 ***	−3.54 **	−3.55 ***	−1.22	−1.35	−4.40 ***	−4.28 ***
l-fmedc - l-sfg	−4.16 ***	−3.87 ***	−3.51 **	−3.39 ***	−2.21	−2.31	−4.38 ***	−4.17 ***
l-fmedc - r-precun	−4.17 ***	−3.99 ***	−3.65 **	−3.62 ***	−1.1	−1.22	−4.34 ***	−4.29 ***
l-ins - r-mtgtempoc	−3.12 ***	−3.10 ***	−1.88	−2.02	−2.34	−2.36	−2.36	−1.84
l-fmedc - r-mtgant	−4.33 ***	−3.95 ***	−2.76 *	−2.45	−1.69	−1.47	−3.26 ***	−3.20 ***
l-ang - l-mtgant	−3.76 ***	−3.91 ***	−3.48 **	−3.68 ***	−3.02 *	−3.14 *	−4.29 ***	−4.26 ***
l-parhipc - r-sfg	−2.79 ***	−2.63 ***	−2.81 *	−2.51	−0.67	−0.89	−4.26 ***	−3.87 ***
l-fmedc - l-parcing	−3.93 ***	−3.62 ***	−3.59 **	−3.45 ***	−2.74 *	−2.74 *	−4.23 ***	−4.05 ***
r-sfg - r-parhipc	−3.03 ***	−3.59 ***	−3.47 **	−3.66 ***	−1.14	−1.34	−4.23 ***	−4.13 ***
l-occfus - l-tfus	−2.66 ***	−2.67 ***	−3.20 *	−3.14 *	−2.16	−2.3	−4.23 ***	−4.18 ***
l-fmedc - l-front	−4.23 ***	−3.49 ***	−3.33 *	−2.73 *	−1.83	−1.43	−3.41 ***	−2.79 ***
l-temp - r-sfg	−2.90 ***	−2.99 ***	−2.56	−2.44	−0.79	−0.78	−4.21 ***	−3.88 ***
r-occfus - r-tfus	−3.74 ***	−4.21 ***	−3.15 *	−3.49 ***	−2.14	−2.3	−3.66 ***	−3.57 ***

The overall 30 most significant autism-related group differences in order of significance level. The first column lists the short names of the region pairs (See Table 1 for full names and other information). The corresponding T-value estimated from the STD and STD+RDI models are shown for all investigated nuisance signal regression methods. Significance level is implied by asterisks: * p<0.01, ** p<0.001, *** FDR q<0.05.

**Table 5:** Autism-related group differences (with nuisance signal regression of 6 motion parameters).

Connection	NOREG+M6	+RDI	WMCSF+M6	+RDI	GSREG+M6	+RDI	COMPC+M6	+RDI	SAT36	+RDI
l-cun - l-stg	−2.88 *	−2.6	−2.54	−2.29	−2.93 *	−2.41	−3.57 **	−3.13 *	−2.14	−1.52
l-occfus - r-tfus	−1.98	−2.05	−2.16	−2.25	−2.33	−2.3	−2.96 *	−2.84 *	−2.35	−2.4
l-fmedc - r-parcing	−4.18 ***	−4.01 **	−3.98 **	−3.90 **	−2.82 *	−3.08 *	−3.86 **	−4.00 **	−2.24	−2.82 *
l-fmedc - r-cingpost	−4.61 ***	−4.31 ***	−4.63 ***	−4.54 ***	−2.93 *	−3.12 *	−4.21 ***	−4.16 **	−2.38	−2.63 *
r-mfg - r-parhipc	−1.55	−1.62	−2.25	−2.36	−1.35	−1.56	−1.8	−1.63	−1.21	−1.28
l-smargp - l-mtgant	−1.87	−1.99	−1.78	−1.97	−2.55	−2.65 *	−2.88 *	−2.86 *	−2.75 *	−2.82 *
r-tfus - r-tofus	−1.6	−2.1	−1.63	−2.18	−1.05	−1.5	−2.5	−2.95 *	−1.24	−1.66
l-fmedc - l-cingpost	−4.58 ***	−4.31 ***	−4.56 ***	−4.51 ***	−2.73 *	−3.01 *	−4.24 ***	−4.25 **	−2.27	−2.49
l-hip - l-thal	0.56	0.8	1.44	1.43	2.54	2.48	1.16	1.7	2.28	2.19
l-sfg - l-mtgant	−2.52	−2.58	−2.59	−2.75 *	−3.41 **	−3.41 **	−4.09 **	−4.13 **	−3.84 **	−3.83 **
l-fmedc - r-front	−3.89 **	−3.39 **	−4.04 **	−3.54 **	−2.15	−1.87	−3.23 *	−3.00 *	−2.09	−1.93
l-cun - l-plan	−2.56	−2.24	−2.13	−1.83	−2.06	−1.51	−3.10 *	−2.62 *	−1.28	−0.64
l-stg - r-cun	−2.61 *	−2.39	−2.34	−2.15	−3.15 *	−2.82 *	−3.40 **	−2.99 *	−2.5	−2.03
l-temp - l-thal	1.02	1.28	1.57	1.8	2.53	2.28	0.85	0.95	2.36	2.16
r-parhipc - r-hip	1.6	1.27	1.58	1.32	3.16 *	2.89 *	1.34	1.09	2.77 *	2.6
l-mtgant - r-sfg	−2	−1.99	−2.1	−2.17	−1.25	−1.23	−3.21 *	−3.10 *	−2.2	−2.15
r-fmedc - r-precun	−3.22 *	−3.05 *	−3.16 *	−3.03 *	−1.49	−1.56	−3.25 *	−3.35 *	−1.26	−1.38
l-fmedc - l-precun	−4.40 ***	−4.17 ***	−4.17 ***	−4.09 **	−2.52	−2.61	−4.37 ***	−4.32 **	−2.16	−2.2
l-fmedc - l-sfg	−3.98 ***	−3.73 **	−3.79 **	−3.54 **	−2.63 *	−2.64 *	−3.87 **	−3.77 **	−2.3	−2.14
l-fmedc - r-precun	−4.17 ***	−3.89 **	−4.02 **	−3.85 **	−2.19	−2.24	−3.91 **	−3.86 **	−2.02	−2.02
l-ins - r-mtgtempoc	−2.52	−2.58	−2.18	−2.56	−3.46 **	−3.86 **	−1.77	−1.64	−3.77 **	−4.33 **
l-fmedc - r-mtgant	−3.31 *	−3.25 *	−3.15 *	−3.27 *	−1.71	−2.05	−3.22 *	−3.49 **	−1.68	−1.77
l-ang - l-mtgant	−2.55	−2.71 *	−2.42	−2.67 *	−2.31	−2.58	−2.78 *	−2.88 *	−2.54	−2.83 *
l-parhipc - r-sfg	−2.19	−1.94	−2.24	−2.02	0.31	0.14	−2.85 *	−2.52	0.11	−0.15
l-fmedc - l-parcing	−4.03 ***	−3.81 **	−3.92 **	−3.77 **	−2.81 *	−2.90 *	−3.66 **	−3.72 **	−2.47	−2.80 *
r-sfg - r-parhipc	−1.93	−2.09	−2.2	−2.39	−0.06	−0.32	−2.80 *	−2.65 *	−0.37	−0.53
l-occfus - l-tfus	−1.66	−1.62	−1.78	−1.8	−1.08	−1.28	−2.13	−2.11	−0.75	−0.94
l-fmedc - l-front	−3.53 **	−2.93 *	−3.37 **	−2.66 *	−1.62	−1.09	−3.01 *	−2.39	−1.69	−1.22
l-temp - r-sfg	−1.84	−1.7	−1.98	−1.92	−0.32	−0.26	−3.57 **	−3.38 **	−1.26	−1.2
r-occfus - r-tfus	−1.97	−2.24	−2.38	−2.67 *	−2.19	−2.54	−3.01 *	−3.14 *	−1.89	−2.19

More than 200 connectivity differences survived the q<0.05 false discovery rate criterion in NOREG and COMPCOR (both with and without RDI) and none survived in GSREG+RDI, GSREG+M6, GSREG+M6+RDI, SAT36 and SAT36+RDI. As predicted above, the inclusion of RDI introduces only slight changes in the pattern of autism-related group differences.

All the evaluated signal regression approaches revealed presumably autism-linked impairments of functional connectivity. Autism was mainly characterized by decreased synchronicity, i.e., under-connectivity. This finding is in line with the majority of intrinsic functional connectivity studies. The spatial predisposition, along with the 30 most significant differences, is presented in Fig. 9 and Table 4 and 5.

While including RDI significantly reduced (presumably artifactual) differences between voxel-wise displacement-related subject cohorts, differences in the autism-related comparison were more or less preserved. These results suggest that the proposed correction method, while effectively reducing motion artifacts in group comparisons, preserves the sensitivity to neural differences.

A critical interpretation of our autism-linked findings in this study is not directly possible. This mainly stems from the lack of ground truth information about the basic neuropathology of the disease. Furthermore, larger-scale, multi-centric comparisons would be optimal to test the reproducibility of any finding.

### Contrasting the impact of head motion and nuisance signal regression strategies

The efficiency of nuisance signal regression techniques in the context of rs-fcMRI motion artifacts analysis has been intensively investigated in the last few years; however, a significant part of these studies did not apply second-level regression covariates [2], [3], [9], [16] in population-level analysis. Their overall conclusion was that global signal regression, high-parameter nuisance signal regression, scrubbing, and de-spiking are potentially beneficial. However, recent studies have questioned some of these conclusions. In the following, we summarize the latest findings in the literature in contrast to our results.

#### Effect of global signal regression

In our analysis, patterns of group differences become extremely different when regressing out the whole-brain signal from regional BOLD time courses (GSREG, GSREG+M6 and SAT36). Most of the above-mentioned studies, which did not utilize second-level correction, applied GSREG in their analysis pipeline. Recent studies using mean FD as a second-level regressor [14], [45] also concluded that GSREG mitigates the effects of motion-related differences among subjects, but warns that investigators must weigh up the pros and cons of GSREG when deciding whether to employ it in the context of testing specific hypotheses.

Our results show that, after the correction of group-wise differences in head movement in the autism-control comparison, over-connectivity and under-connectivity can simultaneously appear. However, global signal regression in the processing pipeline appears to bias results toward over-connected differential networks. In addition to under-connectivity, many authors suggested short-distance over-connectivity (in the frontal lobe or globally) as a possible finding in autism spectrum disorders [46]. This theory was then questioned on the grounds that head-movement could induce similar effects [21]. As reported in [47], correlation estimates obtained after GSREG are more susceptible to the presence of motion and exacerbate distance-dependent bias. Moreover, as reported in [20], [22], correlation patterns and group differences may become distorted after GSREG (depending on, e.g., region size or the underlying true connectivity structure). According to Müller et al. [48], pre-processing strategies greatly affect the spatial patterns of autism-linked connectivity traits, although under-connectivity is the most prevalent across studies. It is, therefore, safe to conclude that GSREG not only introduces anti-correlations in the functional connectome [49], but can also confound case-control comparisons in autism.

#### Effect of including motion parameters

In the case of voxel-wise motion pattern-related group comparisons, inclusion of the six motion parameters in first-level nuisance signal regression showed no obvious improvement in motion-artifact reduction. However, the inclusion of these parameters had a pronounced effect in autism-related group comparisons, especially in NOREG, WMCSF and COMPCOR techniques: the number of significant group differences decreased. Whether this phenomenon is due to improved motion-artifact reduction in the special case of autistic group comparisons, or due to overfitting and the removal of real brain signal, remains a question. However, evidence that spatial realignment-based estimation of motion parameters may yield poor results in periods of small movements [12] point toward the conclusion that motion parameter estimates should be applied carefully. As a possible solution, we suggest utilizing thresholded motion estimates and avoiding the use of motion parameters in periods of relatively small movements.

#### Optimal choice of individual-level motion-correction technique

In some cases, it is not clear whether various high-parameter nuisance regression techniques eliminate group differences due to increased specificity or decreased sensitivity. Considering most significant autism-related group differences, minimal nuisance regression techniques (NOREG, NOREG+M6) show a high consensus with moderate (WMCSF, WMSCF+M6) and more complex (COMPCOR, COMPCOR+M6) methods. This points to the conclusion that when analyzing a sufficiently large sample and utilizing an appropriate second-level model, the choice of individual-level signal nuisance regression technique becomes less crucial. However, when analyzing small samples and also with individual analysis, the role of these techniques is unquestionably important.

#### The role of confounds not related to subject motion

This article focused on motion-related artifacts, which are only one, although a conspicuous source of confounding effects in functional MRI. In thes context, the performance of NOREG and NOREG+M6 methods in motion-related comparisons deserves attention. One possible explanation is that, although artifacts of other sources are obviously present in the data, their individual spatio-temporal pattern is more constant and their population-level distribution is more similar among the investigated subpopulations compared to motion-related confounds. Thus, these artifacts may have only a moderate disturbing effect in large-sample group comparisons. However, by grouping conditions in relation to physiological conditions, like blood pressure and blood oxygenation (or artifacts of scanner-related sources in multi-center studies), non-motion originated artifacts can appreciably affect the results. Thus in such experimental designs, nuisance regression methods may have a more important role.

This is also suggested by the results of autism-related group comparisons, where pronounced differences were experienced among various nuisance signal regression techniques. A potential explanation for these deviations is that, although the proposed motion-correction technique successfully reduces motion-related erroneous group differences, artifacts of other physiological and scanner-related sources affect autism-related group comparisons and these phenomenon is handled differently by various signal regression techniques.

## Conclusion

In this study, we demonstrated that small movements during scanning can cause different displacements in various locations of the brain, and, accordingly, motion-related BOLD signal changes also depend on location. We characterized the effect of this spatio-temporally complex BOLD artifact pattern on functional connectivity. We proposed RDI, a set of regression covariates for the population-level correction of motion artifacts arising from local head motion. As shown with comparisons of groups with differing average voxel-wise motion pattern, the proposed correction technique efficiently reduces artifacts caused by differences in voxel-wise motion patterns in population-based connectivity analysis; and meanwhile, as demonstrated by comparing autistic and control groups, preserves differences corresponding to neural origin. Our findings suggest that, especially by moderate nuisance correction methods, the inclusion of RDI as second-level nuisance covariates is generally appropriate and may become increasingly necessary when the variable of interest is interrelated with altered subject kinetics.

A limitation of the proposed method is that it cannot be effectively applied in case of individual studies or small sample sizes. Nevertheless, one should note that, in situations where the variable of interest is correlated with motion, second-level regression-based, motion-correction approaches can be conservative, as they remove common variation among regressors. Furthermore, the proposed method is based only on a simplified measure of motion and does not handle rapid sub-TR displacements, which may play an important role in regional motion artifact interactions.

The question of what is the optimal individual-level signal regression technique for motion correction remains open, but, seems less crucial for large-sample, group-level studies using a proper second-level correction method.

This article focused on motion-related artifacts, which are only one, although a conspicuous source, of confounding effects in functional MRI. In-scanner head motion is relatively easy to measure, and thus, corresponding artifacts are actively investigated. However, as suggested by the observed differences among nuisance signal regression techniques, physiological and scanner-related artifacts may also have an essential impact on fc-MRI studies.
